# CLEC5A Activation in Inflammatory Monocytes: A Mechanism for Enhanced Adaptive Immunity Following COVID-19 mRNA Vaccination in a Preclinical Study

**DOI:** 10.3390/v17091233

**Published:** 2025-09-10

**Authors:** Renan Galuzo, Thiago Lazari Machado, Ryann de Souza Nascimento, Jorvan Ramos de Medeiros, Luciana Neves Tubarão, Jane Silva, Vanessa Pimenta Rocha, Tamiris Azamor, Felipe Soares Coelho, Andrea Marques Vieira da Silva, Lorenna Carvalho da Rosa, Juliana Fernandes Amorim da Silva, Renata Tourinho Santos, Rodrigo Müller, Carolina Baeta Salvador Várady, Ana Paula Dinis Ano Bom, Patricia Cristina da Costa Neves, Juliana Gil Melgaço

**Affiliations:** 1 Instituto de Tecnologia em Imunobiológicos, Bio-Manguinhos, Fundação Oswaldo Cruz, FIOCRUZ, Rio de Janeiro 21040-900, Braziljorvan.medeiros@bio.fiocruz.br (J.R.d.M.); juliana.silva@bio.fiocruz.br (J.F.A.d.S.); rmuller@bio.fiocruz.br (R.M.); carolina.varady@bio.fiocruz.br (C.B.S.V.); pcristina@bio.fiocruz.br (P.C.d.C.N.); 2 Biological Sciences Building, Kensington Campus, University of New South Wales, Sydney, NSW 2033, Australia

**Keywords:** mRNA vaccine, animal models, SARS-CoV-2, CLEC5A, immunity

## Abstract

Background: CLEC5A is a C-type lectin expressed by monocytes and neutrophils, playing an important role in innate immunity. Although it has been shown to interact with the spike protein of SARS-CoV-2, its role during vaccination remains poorly understood. Methods: To address this question, we combined in vitro assays to characterize CLEC5A and spike expression and their impact on monocyte differentiation and T-cell activation; in vivo studies to evaluate CLEC5A expression, immune responses, and vaccine efficacy in a murine model; and in silico analyses to identify potential spike epitopes and CLEC5A interaction sites. Results: The Pfizer-BioNTech bivalent mRNA vaccine induced spike expression and CLEC5A upregulation in THP-1 monocytes, promoting M1-like differentiation and CD86^+^ activation. In PBMC co-cultures, CLEC5A^+^ monocytes acted as antigen-presenting cells, releasing inflammatory chemokines and activating both CD4^+^ and CD8^+^ T cells, thereby linking CLEC5A expression to adaptive immunity. In mice, CLEC5A expression was observed on inflammatory monocytes (CCR2^+^CX3CR1^low^) within two days of vaccination. In vivo, CLEC5A expression increased during SARS-CoV-2 infection and after immunization, but declined following viral challenge in vaccinated animals. Consistently, robust humoral and cellular responses were detected post-immunization. In silico analysis further suggested differential CLEC5A binding across B- and T-cell epitopes within the spike glycoprotein. Conclusions: These findings suggest that CLEC5A may play a role in bridging innate and adaptive immune responses during SARS-CoV-2 vaccination. Although further studies with different vaccine platforms are necessary to confirm and expand these observations, our results provide preliminary evidence supporting the potential of CLEC5A as an exploratory biomarker of vaccine-induced immunity.

## 1. Introduction

The COVID-19 pandemic, caused by SARS-CoV-2, led to high morbidity and mortality rates in 2020–2021, prompting a global effort to develop treatments and vaccines [1]. Various vaccine platforms, including viral vectors and mRNA vaccines, were successfully developed and widely licensed by 2021 [2]. While these vaccines significantly reduced severe cases and deaths, emerging variants like Omicron posed challenges due to increased transmissibility and immune evasion [3].

To ensure long-term immunity, it is crucial to evaluate the persistence of memory cells, cross-reactivity among variants, and the effectiveness of bivalent vaccines in preventing severe cases. Inflammation control remains a key factor in disease regulation post-vaccination, as SARS-CoV-2 affects systemic immune homeostasis and vaccine functionality [4,5,6,7,8].

Given that inflammation control is a crucial determinant of vaccine-induced immunity, receptors involved in pathogen recognition and inflammatory signaling, such as CLEC5A, have gained increasing attention [4,5,6,7,8].

CLEC5A, also known as myeloid DAP12-associating lectin-1 (MDL-1), is a Syk-coupled pattern recognition receptor predominantly expressed on monocytes, macrophages, and neutrophils. It preferentially recognizes glycans that are abundantly displayed on the surface of viruses and other pathogens. Upon antigen binding, CLEC5A can form multivalent heterocomplexes with other C-type lectins, such as DC-SIGN and the mannose receptor, as well as with Toll-like receptors (TLRs). Then, an inflammatory cascade of cytokines can be produced by myeloid cells, leading to the activation of innate immunity, followed by an adaptive immune response of CD4+ T-cell activation, antibody production, as well as cytotoxic CD8+ T-cell profile, increasing the antiviral response and promoting long-term immunity, as seen in the yellow fever virus attenuated vaccine [9,10].

In our previous study with SARS-CoV-2 infection, we demonstrated that exacerbated expression of CLEC5A on inflammatory monocytes plays a role in the innate response triggered by SARS-CoV-2 proteins, inducing poor outcomes [11]. However, their role in COVID-19 vaccination remains underexplored. Further research is needed to clarify CLEC5A’s role in SARS-CoV-2 immunity and its potential impact on mRNA vaccine efficacy [9].

In light of the limited knowledge about the role of the CLEC5A receptor in the immune response induced by mRNA vaccines against SARS-CoV-2, the aim of this study was to address this gap using preclinical approaches: (a) in vitro, by investigating CLEC5A and spike protein expression in the human monocytic cell line THP-1, establishing a kinetic profile to evaluate inflammatory markers and the potential for differentiation into antigen-presenting cells capable of activating T lymphocytes in co-culture systems; (b) in vivo, by assessing CLEC5A expression in a suitable animal model for mRNA vaccine studies targeting SARS-CoV-2, with evaluation of classical features of innate and adaptive immune activation, including immunogenicity and viral challenge protection; and (c) in silico, by analyzing the amino acid sequence of the spike glycoprotein to identify potential interaction sites with CLEC5A and epitopes involved in B- and T-cell responses in a murine model.

## 2. Methods

### 2.1. Monocyte Cell Line THP-1 In Vitro Assays

THP-1 cell line is a human monocytic leukemia cell line (THP-1—TIB-202, ATCC) used in the in vitro assays to verify any differentiation caused by the bivalent commercial COVID-19 mRNA vaccine (Pfizer BioNTech^®^, Cambridge, MA, USA). The THP-1 cells were transfected with the vaccine, assisted by Opti-MEM I reduced-serum medium without antibiotics (Opti-MEM™ I Reduced Serum Medium—CAT: 31985062—Thermo Fisher Scientific, Waltham, MA, USA) and incubated for 1, 2, and 3 days. This was based on the activation peak reported in the literature [12].

After establishing the peak of spike expression, the THP-1 cells were incubated with mRNA vaccine and lipofectamine, as vehicle-only control (mock) (Lipofectamine 2000, ThermoFisher, Waltham, MA, USA, Cat#11668019) to investigate whether the cells were able to express CD11b or CD38 as markers of transitioning to macrophage-M1 phenotype, as well as to antigen-presenting cells with CD86+ expression in the same time points (1, 2, and 3 days) [13,14,15].

As soon as the best time points were chosen, the transfected THP-1 was co-cultured with peripheral blood mononuclear cells (PBMC) in the ratio 1:10 (PBMC:THP-1) from vaccinated subjects without previous SARS-CoV-2 infection (30 days after second dose) to verify whether CD4+ and CD8+ T lymphocytes would be activated through the antigen presenting cell differentiation induced by the mRNA vaccine in the THP-1 [16]. The variety of T-cell phenotypes was assessed through flow cytometry, and the list of antibodies used is available in Supplementary Table S3 [17].

Five human PBMC samples from vaccinated subjects were from our previous study, which the samples were frozen in liquid nitrogen and thawed for this assay. All procedures for obtaining samples were approved by the Ethics Committee of Fiocruz (Protocol# 34728920.4.0000.5262) in accordance with the Helsinki Declaration as revised in 2013 [14]. Informed consent was obtained from all participants, and all methods were carried out in accordance with relevant regulations and guidelines [18].

Inflammatory cytokines MIP1a, MCP1, IL-8, IL-6, and IL-1b were measured from supernatant of transfected THP-1 cells at the optimal time point before incubation with human PBMC samples. An in-house multiplex liquid microarray test was performed using 106 xMAP microspheres (Luminex Corporation, Austin, TX, USA) which were coupled with anti-human purified mouse monoclonal antibodies at the following concentrations: anti–IL-6 at 100 μg/mL, anti-MIP1a at 500 μg/mL, anti-IL-8 at 50 μg/mL, anti-MCP1 at 50 µg/mL and anti-IL-1b at 50 µg/mL. Coupling reactions were performed using the Amine Coupling Kit (Bio-Rad) following the manufacturer’s instructions [11,19].

### 2.2. In Vitro Assay of Murine Blood Cells Exposed to the COVID-19 mRNA Vaccine

Blood collected from five mice were treated with ACK lyse solution, as recommended by manufacturer (ThermoFisher, Framingham, MA, USA, cat#A1049201) and the cells were transiently transfected with the COVID-19 mRNA bivalent vaccine from Pfizer BioNTech^®^ (5 µg mRNA/1000 µL, Lot. PAA194703) using a transfection medium. After 1, 2 and 3 days of mRNA vaccine incubation, the cells were washed, collected, and resuspended in fluorescence-activated cell sorting (FACS) buffer (1 × PBS, 2% FBS, 5% horse serum, 0.05% sodium azide). The cells were stained with Live Dead Blue dye for 10 min, and then washed with FACS buffer and centrifuged (500× *g*, 5 min). After that, the cells were stained with anti-mouse antibodies purchased from BD Biosciences (Bedford, MA, USA) and R&D Systems as described by Supplementary Table S3. All antibodies were incubated for 25 min (2–8 °C). The cells were washed and then fixed with 1% PFA. Data acquisition was performed on a BD LSR Fortessa instrument (BD Biosciences) and analyzed using FlowJo software v.10.

### 2.3. Fluorescence Microscopy of the THP-1 Cell Line

To confirm the surface expression of the spike protein, THP-1 cells were incubated with the mRNA vaccine in a 5% CO_2_ incubator at 37 °C. After incubation, the cells were fixed with 4% paraformaldehyde, washed with blocking buffer (PBS supplemented with 5% FBS and 2% horse serum), and stained with anti-CD11a to identify the cell surface (Supplementary Table S3). The cells were then treated with 0.1% Triton-X permeabilization solution. Following permeabilization, the samples were stained with anti-spike antibody to evaluate the cellular localization of the spike protein. Images were acquired at 400× magnification using the EVOS M7000 Imaging System.

### 2.4. Animal Study Design

Adult mice (*Mus musculus*) K18-hACE2 lineage weighing 20–30 g and 8–10 weeks old were used as an animal model for this study. Of the 60 mice, 40 were female. The study protocol was also approved by Fundação Oswaldo Cruz animal ethics committee (LW-17/20). All procedures involving animals were conducted in Animal Biosafety Level 3 at the Preclinical Testing Laboratory (LAEPC) of the Department of Experimental Development and Preclinical Research at Bio-Manguinhos/Fiocruz and we confirm that all experiments were performed in accordance with relevant guidelines and regulations.

The mice were divided into four groups (*n* = 15 mice/group): 1- control group receiving saline buffer (Tris HCl pH 7.4, 100 µL), 2- animals who received two doses of COVID-19 vaccine, 3- animals who were virally challenged and not vaccinated, 4- animals who were vaccinated with two doses and were virally challenged. The animals were followed through 14 days after viral challenge until euthanasia, and every day the weight was measured, and clinical signs were monitored until severity for humane endpoint (e.g., piloerection, arched back, respiratory distress, ocular discharge and lethargy) or euthanasia day (14th day after SARS-CoV-2 challenge). The SARS-CoV-2 RT-qPCR was performed after challenged and immunization using oropharyngeal swabs from third- and fifth-days post-infection (DPI), and from lung and brain sections extracted after euthanasia on days 5, 6, and 14 post infection.

A COVID-19 mRNA bivalent vaccine from Pfizer BioNTech^®^ (30 µg mRNA/300 µL, Lot. PAA194703) was used for immunization. The animals were contained for intramuscular immunization at 1/6 of the human adult dose (50 µL per hind limb). Two doses were administered, with an interval of 28 days between the first and second dose. For in vitro study, five animals (non-immunized; male: 3) were used.

The animals were anesthetized via inhalation using up to 1.5–3% isoflurane (dose-dependent) in an acrylic anesthesia chamber specifically designed for laboratory animals (INHALATION ANESTHESIA EQUIPMENT BY INFUSION—Bonther^®^, Ribeirão Preto, SP, Brazil). After complete relaxation, the animals were kept in an upright position for intranasal inoculation. The Gamma variant (P.1) of SARS-CoV-2 was intranasally inoculated with 1 × 10^5^ PFU in 10 µL (5 µL per nostril) using a 2–20 µL automatic pipette (Rainin^®^, Sigma-Aldrich, St. Louis, MO, USA) at the 14th day after the second dose of COVID-19 vaccine. The same anesthesia procedure was applied on the 3rd and 5th DPI for swab sample collection of oropharyngeal cavities.

Euthanasia was performed one day before viral challenge (at day 13 post 2nd dose of vaccination) and in specific euthanasia days (5th, 6th and 14th day after SARS-CoV-2 challenge) through anesthesia with doses of 200 mg/kg of ketamine hydrochloride and 20 mg/kg of xylazine hydrochloride administered intramuscularly. After several minutes of anesthetic administration and the absence of palpebral and interdigital reflexes (tested by touch), blood sample collection by cardiac puncture was performed. Next, sodium thiopental was administered intraperitoneally at a dose of 150 mg/kg for spleen, lung, and brain sample collection.

The adult mice (*Mus musculus*) K18-hACE2 lineage were purchased from The Jackson Laboratory (Bar Harbor, ME, USA), maintained by the Institute of Science and Technology in Biomodels (ICTB)/Fiocruz and housed at a density of five animals per cage, with weights varied to ensure heterogeneity within each group. The airtight cages measured 36 cm in length, 17 cm in width, and 15 cm in height, and were set up in a ventilated microisolator rack system (ALESCO^®^, Fort Myers, FL, USA) using high negative pressure system (ALN 2) with autoclavable wood shavings as substrate. Water and food were changed twice a week under unrestricted access conditions. The mice were maintained under a 12-h light/12-h dark cycle in a controlled environment with temperatures ranging from 18 to 24 °C, relative humidity between 40 and 70%, and a constant air exchange rate of 20 changes per hour.

The experimental laboratory has an Environmental Enrichment Program that includes a polysulfone igloo as a shelter, with cotton rolls, shredded paper, and cardboard being interchanges monthly as forms of environmental enrichment. Clinical evaluations of the animals were conducted by a veterinarian based on the ARRIVE guidelines, and humane endpoints were established based on the severity of clinical signs.

The health monitoring of the colony providing the animals was conducted quarterly based on a specific pathogen list, as recommended by the Federation for Laboratory Animal Science Associations (FELASA), with all animals included in this study considered free of specific pathogens (SPF).

### 2.5. Gene Expression for CLEC5A and Innate Immunity-Related Genes

**Figure 1:**
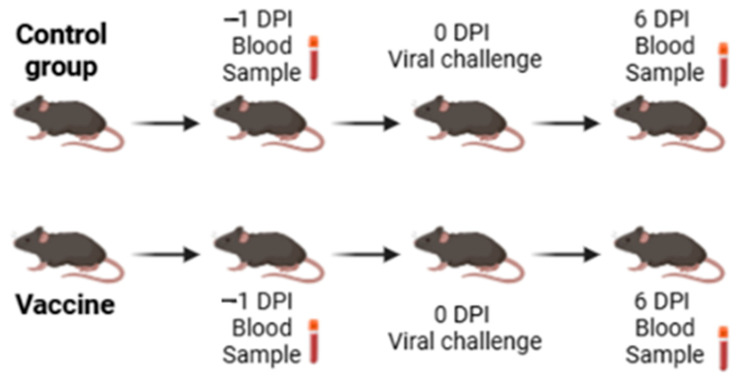
Experimental model of gene expression. The experimental model assay of gene expression is composed of the vaccine group and control group. For this assay, blood collection was performed twice: one day before the viral challenge and six days post-viral challenge in both groups.

To evaluate the gene expression after the vaccination and the viral challenge, total RNA was obtained from blood samples from 60 animals using the TRIzol extraction method (Invitrogen, Carlsbad, CAM, USA). RNA mass was quantified using a spectrophotometer (260 nm; Nanodrop Technologies, Wilmington, DE, USA), followed by a complementary DNA (cDNA) synthesis from 250 ng of total RNA using the High-Capacity cDNA Reverse-Transcription Kit (Thermo Fisher Scientific, Framingham, MA, USA), with both procedures carried out according to the manufacturer’s instructions. The experimental design for analyzing gene expression is illustrated in Figure 1, with the timing of sample collection.

For the quantitative RT-qPCR reaction, Fast Sybr Green master mix (Applied Biosystems, Foster City, CA, USA) with 200 nM of each primer (Supplementary Table S1) and 10 ng of each cDNA were used in a final reaction volume of 10 µL. QuantStudio 7 PRO (Applied Biosystems, Foster City, CA, USA) instrument was used to calculate the Ct values during the RT-qPCR assay and the cycling conditions involved: polymerase activation at 95 °C for 20 s; followed by up to 40 cycles of denaturation at 95 °C for 15 s and annealing/extension at 60 °C for 1 min.

The data obtained were normalized to the average cycling threshold of the reference genes PPIA and GAPDH and expressed as fold change (2-ΔΔCt) using the control group receiving saline buffer as a reference for calibration.

A sub-analysis using total RNA from blood samples of 15 animals was conducted to characterize the transcriptomic profile of a panel of innate-immunity-related genes. To test this panel, we used 96.96 gene expression Integrated Fluidic Circuit for Biomark HD platform (99.96 GE IFC, Fluidigm, #BMK M-96.96).

The cDNA from each of the 96 samples was synthesized from 250 ng of RNA using Reverse Transcription Master Mix (Fluidigm, South San Francisco, CA, USA, #100-6297). Afterward, the cDNAs were simultaneously preamplified with a pool of all primer pairs in a conventional thermocycler. To this end, we used Preamp Master mix (Fluidigm #1005581) with 500 nM of each primer (forward and reverse) and 1.25 µL of each cDNA in a final reaction volume of 5 µL for 14 cycles. Preamplified cDNA was treated with Exonuclease I (New England BioLabs, MA, USA, # M0293S), and diluted 1:5 in TE Buffer pH 8.0 (10 mM Tris-HCl, 0.1 mM EDTA). The preamplified cDNAs mixed with 2x SsoEvaGreen Supermix with low ROX (Bio-Rad, Hercules, CA, USA, #64461016) as well as 20 nM of individual primer pairs were loaded in the 99.96 GE IFC using the Juno system (Fluidigm). Real-time PCR reactions were conducted in the Biomark HD microfluidic system (Fluidigm). The melting curve of each primer pair and sample quality were analyzed on the company’s software and unexpected values were considered for primer or sample exclusion.

After quality control exclusions, a panel of 78 genes was analyzed including genes related to innate immunity and the references B2M, GAPDH, and ACTB (Supplementary Table S2). The data obtained were normalized to the average cycling threshold of the reference genes and expressed as fold change (2-ΔΔCt) using the control group receiving saline buffer as a reference for normalization.

### 2.6. Specific Spike-IgG Detection

Microtiter ELISA plates (Maxisorp NUNC—Thermo Fisher Scientific) were coated with 2.5 μg/mL of Spike trimer XBB Omicron (cat.101677) in carbonate/bicarbonate buffer pH 9.6 overnight at 4 °C. Coated plates were washed with PBST (PBS and Tween 20 at 0.05%) and blocked for 1 h at 37 °C with blocking and diluent solution—BDS (PBS, Tween 20% at 0.05%, bovine serum albumin at 0.05%, and skim milk at 5%). The plates were washed again with PBST, then 3-fold serially diluted mice serum samples were added to each well and incubated for 2 h at 37 °C. Serial 3-fold dilutions ranging from 0.007 to 5 µg/mL of mouse anti-spike monoclonal antibodies against SARS-CoV-2 (lot 201112S134SPIKP) were used as standard curve. The plates were washed again before the addition of anti-mouse IgG-peroxidase (cat. A9044—Sigma-Aldrich, Burlington, MA, USA) diluted 1:20,000 in BDS and incubated for 1 h at 37 °C. Finally, the plates were washed before the addition of TMB plus (Kementec Solutions/Bio-Connect Diagnosis BV, Huissen, The Netherlands) for 15 min in the dark at room temperature. The colorimetric reaction was stopped with H_2_SO_4_ 2 M and measured at 450 nm in a plate reader VersaMax™ (Molecular Devices, San Jose, CA, USA). The optical density (OD) of the sera dilutions was plotted on the standard curve, and the antibody titers were calculated by a four-parameter logistic regression using the software SoftMax^®^ (version 7.1.0) (Molecular Devices, San Jose, CA, USA) and expressed as µg/mL.

### 2.7. Polyfunctional Assay for Spike-Specific B- and T-Cell Evaluation

The spleens of the mice were collected and placed in RPMI medium containing 10% FBS and 1% penicillin-streptomycin solution. Splenocytes were isolated into a single-cell suspension by passing the spleens through a 40-μm filter. Erythrocytes were removed using a red blood cell lysis buffer, and the resulting splenocytes were washed, counted, adjusted to 1 × 106 cell/well and exposed to spike peptides at 1 µg/mL (PepMix^TM^ SARS-CoV-2, Spike BA.4/5) and purified αCD28/αCD49d mouse antibodies using a commercial kit for IFN-gamma ELISPOT (Mabtech #3321-4APT-2) as well as in 96-well plates for antibody staining for FACS analysis. The splenocytes with peptides were incubated for 48 h, and then the ELISPOT assay was performed following the commercial kit instructions. The splenocytes for phenotyping assay were washed with FACS buffer and stained with specific antibodies for flow cytometry analysis. The negative control was set up using only cultivation media (RPMI1640 supplemented as described previously). The positive control was concanavalin A at 1 µg/mL. The antibodies were purchased from BD Biosciences, and the list is provided in Supplementary Table S3. After staining assay, the cells were fixed with paraformaldehyde 1%, acquired using LSR Fortessa (BD Biosciences), and analyzed through FlowJo v.10.8 software (BD Biosciences). The gating strategy to identify B and T cells upon spike stimulation is displayed in Supplementary Figure S1.

### 2.8. Molecular Docking to Predict Peptide Epitopes and Assess Residues of Spike Interaction with Immune Cells and CLEC5A

In silico analysis was performed to investigate the peptide epitopes for CLEC5A interaction as well as lymphocyte T and B cell activation through the spike glycoprotein used in the in vitro stimulation assay (Pepmix JPT peptides, PM-WCPV-S-2). IEDB at http://tools.iedb.org/bcell/ (accessed on 13 March 2025) was used to predict linear T-cell and B-cell epitopes in the protein sequence. ClusPro 2.0 server (https://cluspro.bu.edu/login.php (accessed on 15 March 2025)) was used to dock the CLEC5A receptor with spike glycoprotein (PDB: 6VXX). After the docking prediction by Cluspro, the interactions between the CLEC5A receptor and spike protein were predicted through PDBsum server (http://www.ebi.ac.uk/pdbsum (accessed on 5 May 2025)) to obtain residue interactions and bounding information.

### 2.9. Statistical Analysis

Statistical analyses were conducted using GraphPad Prism (version 8.4.2 for Windows). Data normality was assessed with the Kolmogorov–Smirnov or Shapiro–Wilk tests. Group differences were analyzed using Kruskal–Wallis test, and results are presented as mean ± standard error in bar graphs. Statistical significance was set at *p* < 0.05. Final data were obtained from two independent assays using a mouse model and triplicates in the in vitro experiments. In vitro tests were repeated at least one more time to confirm the data. Linear correlation was performed to evaluate the kinetics of protein expression through in vitro assays.

## 3. Results

### 3.1. The Peak of Spike Protein Expression in THP-1 Cells Occurred Two Days After Incubation with the mRNA Vaccine

**Figure 2:**
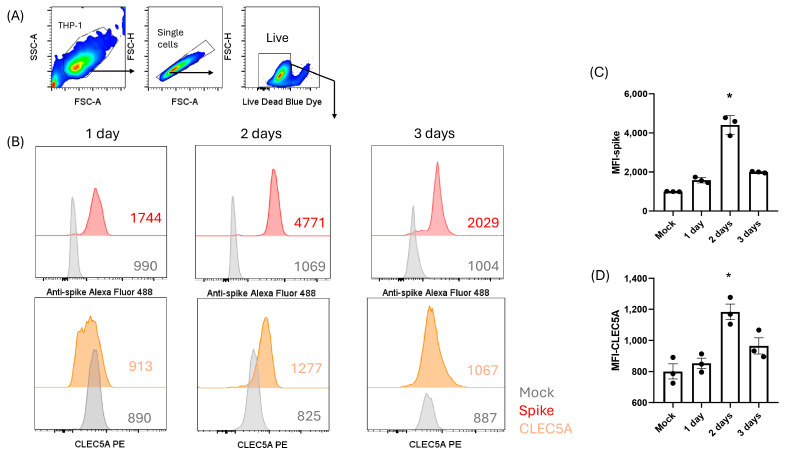
Kinetics of spike protein expression in THP-1 cells by flow cytometry. (**A**) Ancestry gating strategy of the histogram assessing spike expression fluorescence intensity (MFI) in THP-1 cells, showing FSC vs. SSC, followed by singlets and live cells. (**B**) Histogram plot of spike protein and CLEC5A expression in THP-1 cells transfected with the vaccine (red-spike and orange-CLEC5A) compared with THP-1 cells with only culture medium as a negative control (gray), showing MFI values from one of the triplicates. (**C**) Kinetic analysis of spike and (**D**) CLEC5A expression in THP-1 cells, comparing the mean of triplicates using the Kruskal–Wallis test. * *p* < 0.05.

The kinetics of the THP-1 cells incubated with the mRNA vaccine, showed that the highest level of spike and CLEC5A expression was observed at day 2 post-incubation compared with control samples (Kruskal–Wallis test, *p* = 0.013) (Figure 2). A positive linear correlation was found between spike protein and CLEC5A expression (R^2^ = 0.75, *p* = 0.0003).

### 3.2. THP-1 Differentiation in Macrophage-M1-like in the Presence of mRNA Vaccine

**Figure 3:**
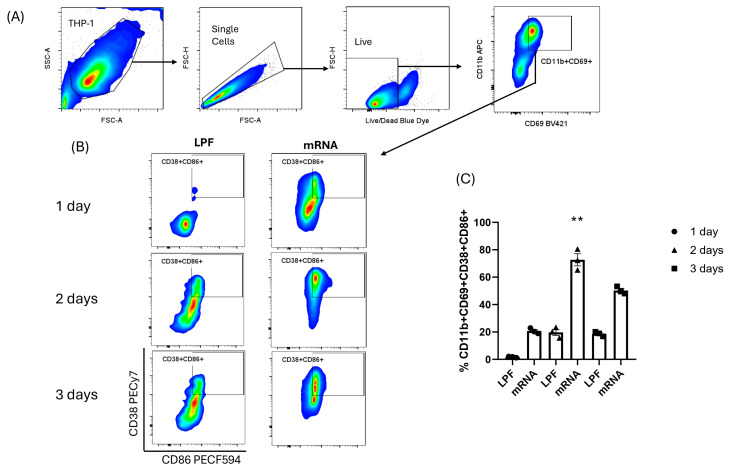
M1-like macrophages are activated in THP-1 cells exposed to the mRNA vaccine. Gate strategy indicated by the black arrow shows THP-1 cells subjected to a time-course incubation with the mRNA vaccine to assess the expression of activated macrophage phenotype markers (CD11b^+^CD69^+^) (**A**) and subsequently the percentage of cells exhibiting an antigen-presenting phenotype (CD38^+^CD86^+^) (**B**). Triplicate assays were analyzed by flow cytometry, with Lipofectamine used as the vehicle-only control (mock), and mean values compared using the Kruskal–Wallis test (**C**). LPF: lipofectamine, mRNA: COVID-19 mRNA, ** *p* < 0.01.

Flow cytometry demonstrated that the mRNA vaccine induced THP-1 activation toward an M1-like macrophage phenotype, with ~60% higher activation than mock (lipofectamine [LPF]) after two days. While vehicle-only (LPF) also promoted activation over time, its effect remained consistently lower than that of the mRNA vaccine at the same time points (Figure 3).

### 3.3. Co-Cultivation of mRNA+THP-1 and Human PBMCs from COVID-19 Vaccinated Subjects

**Figure 4:**
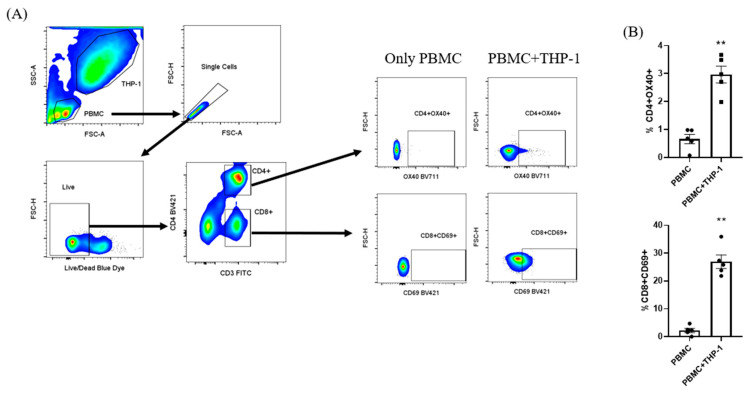
Co-culture of THP-1 cells with PBMCs to assess T cell activation. Sample acquisition was adjusted to define THP-1 and PBMC gates, ensuring that only PBMCs were analyzed for CD4+ and CD8+ T cell subsets (**A**). To evaluate activation, the expression of OX40 and CD69 was assessed in CD4+ and CD8+ T cells, comparing PBMCs co-cultured with THP-1 cells to non–co-cultured controls (**B**). ** *p* < 0.01.

To assess T cell activation induced by antigen-presenting cells during THP-1 differentiation, a 2-day incubation with the mRNA vaccine was performed. PBMCs were then co-cultured with THP-1 cells, and after 24 h the co-culture was resuspended and processed for phenotypic staining by flow cytometry. A significant activation of both CD4+ and CD8+ T cells was observed (Figure 4). In addition, the chemokines MCP-1 (CCL2) and IL-8 (CXCL8) showed 2.07-fold and 4.98-fold increases, respectively, compared with the control at day 2 of THP-1 culture with the mRNA vaccine alone (Kruskal–Wallis test, *p* = 0.03). No significant changes were observed for the other cytokines.

### 3.4. Murine Inflammatory Monocytes with mRNA Vaccine Express CLEC5A Protein

**Figure 5:**
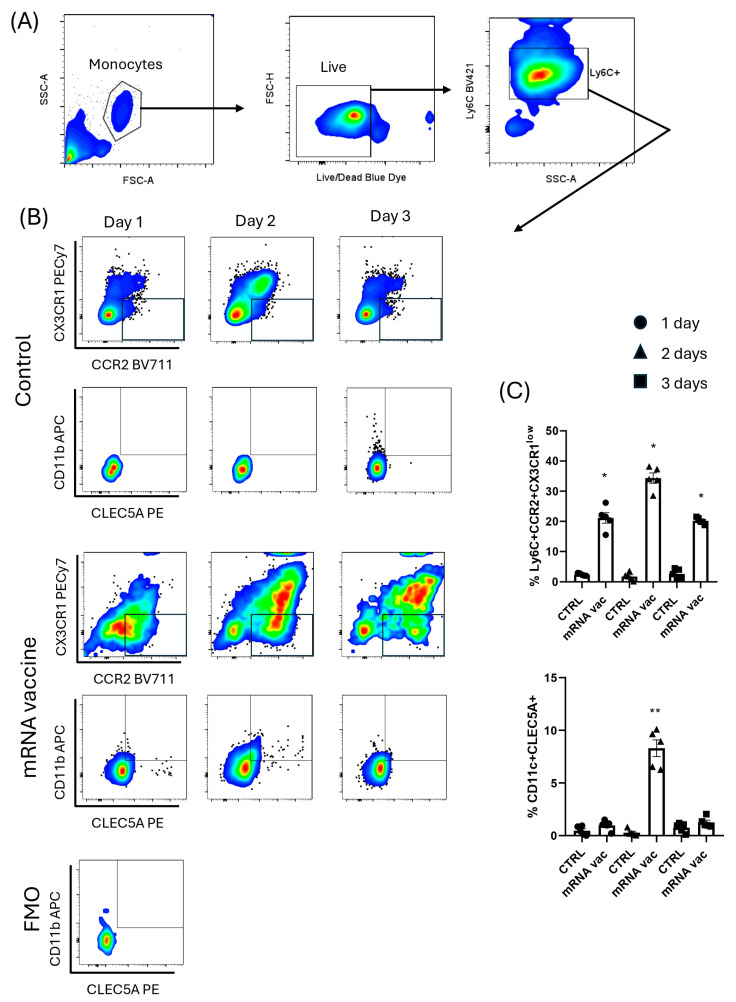
In vitro expression of CLEC5A on inflammatory monocytes from blood of k18-hACE2 mice. Gating strategy to analyze the inflammatory monocytes from blood of mice is indicated by black arrows (**A**). Inflammatory monocytes (Ly6C+CCR2+CCR1^low^) in contact with mRNA vaccine can activate CLEC5A expression on monocyte-derived dendritic cells (CD11c+) represented by dot plot graphs (**B**) and cell expression (%) analyzed by flow cytometry assay (**C**). Fluorescence minus one (FMO) made for CLEC5A-PE marker in the panel, * *p* < 0.05, ** *p* < 0.01.

The cells from murine blood incubated with mRNA vaccine at day 2 showed a significant increase of inflammatory monocytes (Ly6C+CCR2+CX3CR1low) (Figure 5) and a significant increase of CLEC5A co-expressed with CD11c (dendritic like) by those cells (Figure 5) compared with control cells. The gating strategy is displayed in Figure 5.

### 3.5. Fluorescence Microscopy of THP-1 Cells Reveals Spike Protein Expression at the Cell Surface

**Figure 6:**
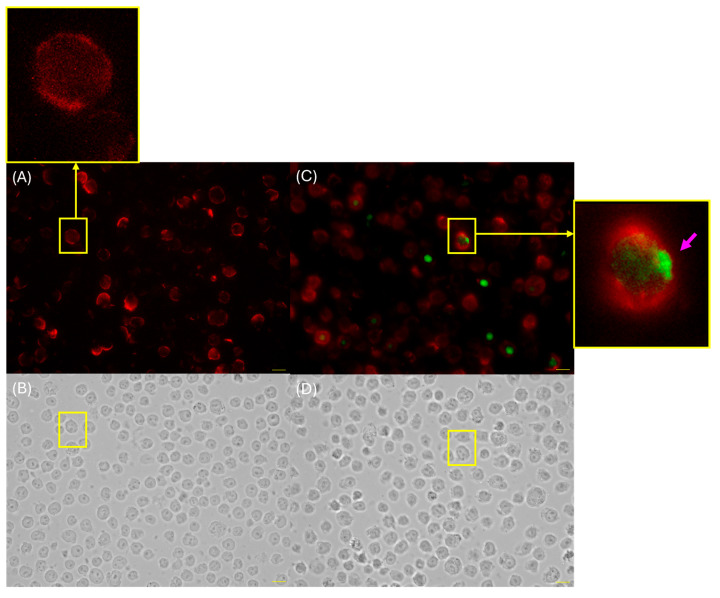
Fluorescence microscopy of spike protein localization in THP-1 cells. THP-1 cells without mRNA vaccine (control) were stained with CD11a-PE and anti-spike Alexa Fluor 488 in dark field (**A**) and bright field (**B**). THP-1 cells were incubated with the mRNA vaccine stained with CD11a-PE and anti-spike Alexa Fluor 488 in dark field (**C**) and bright field (**D**).

As the peak of spike expression was detected at 2 days in flow cytometry assays, fluorescence microscopy was performed at this time point to determine the cellular localization of spike in THP-1 cells. To facilitate membrane identification, an anti-CD11a antibody, commonly expressed on the surface of this cell line, was used. The images demonstrate spike protein expression in THP-1 cells, along with CD11a staining at the cell surface (Figure 6).

### 3.6. In Vivo Experiment: Clinical Characteristics of Animal Model After Immunization and Viral Challenge

**Figure 7:**
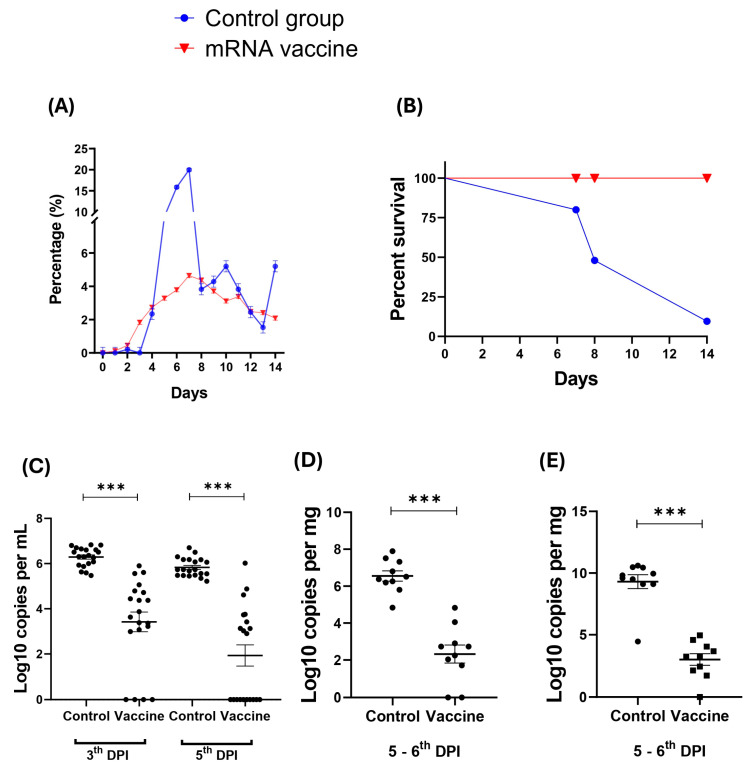
Clinical characteristics and viral load in swab, lung, and brain samples from mice immunized and challenged with SARS-CoV-2. The percentage of weight loss (**A**) and survival (**B**) were monitored for up to 14 days after the viral challenge. After 14 days all animals were humanely euthanized. The viral load post-challenge and immunization was assessed using RT-qPCR from oropharyngeal swabs on the third and fifth days post-infection (DPI) (**C**), as well as from lung (**D**) and brain (**E**) sections extracted after euthanasia on the fifth and sixth-days post-infection. Viral load detection limit: 1 log_10_ copies/mL. *** *p* < 0.001.

The vaccinated group exhibited a maximum weight loss of 4.64% on Day 7 over the 14-day period, while the control group showed a significantly higher weight loss of 19.98% on the same day (Figure 7A). Between Days 7 and 11, a slight difference in weight loss was observed between the control and vaccinated groups, though both groups demonstrated a reduction in weight loss toward the end of the observation period (Figure 7A). The survival curve revealed that the vaccinated group was effectively protected against the viral challenge (Figure 7B). All animals were humanely euthanized after 14 days. The viral load significantly decreased three days after the SARS-CoV-2 challenge with the Gamma strain in the vaccinated group, as determined by oropharyngeal swabs (Figure 7C). In lung (Figure 7D) and brain tissue samples (Figure 7E), a significant reduction in viral load was observed in the vaccinated mice group on days 5–6 post-challenge. By the 8th day post-infection, >50% animals in the control group had died, eliminating the possibility of statistical analysis. The standard curve for viral load quantification was generated using a minigene construct containing the SARS-CoV-2 target sequences N1 and N2, which were cloned into a plasmid.

### 3.7. Gene Expression Evaluation After Immunization and Viral Challenge

**Figure 8:**
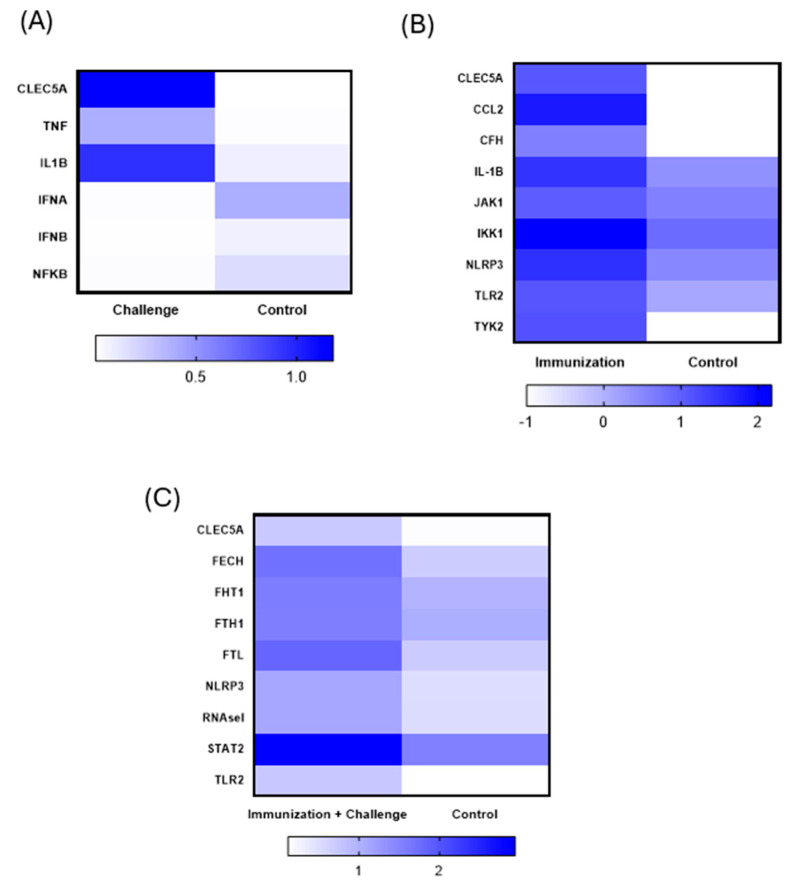
Gene expression profile of mice immunized with bivalent commercial mRNA vaccine. (**A**) Principal component analysis of the gene expression panel of inflammation-related transcripts in blood samples collected 14 days after immunization (*n* = 5) and 14 days after SARS-CoV-2 challenge (*n* = 4) analyzed with the Fluidigm system, in which inflammation-related genes that are significantly altered (*p* < 0.05, unpaired t-test) in mice after (**A**) challenge with SARS-CoV-2 (**B**) immunization and (**C**) immunization + SARS-CoV-2 challenge. Fold change calculated based on relative expression before immunization. Relative expression calculated using average Ct values of *GAPDH*, *ACTB*, and *B2M*.

The gene expression profile of mice after immunization and after immunization + challenge was evaluated using a panel of innate-immune-response related genes, where only significant analysis was plotted (*p* < 0.05) (Figure 8). The genes related to inflammatory profile (IL1B, TNF and CLEC5A) were upregulated in mice infected with SARS-CoV-2, as expected. Meanwhile, the genes related to innate antiviral activity were downregulated (NFKB, IFNA, IFNB) (Figure 8A). The immunization with bivalent mRNA vaccine was characterized by upregulation of genes expressing proinflammatory cytokines (CCL2, and IL1B), complement activation (CFH), inflammasome (NLRP3), and genes involved with receptors of innate immunity that trigger inflammation (TLR2, TYK2, JAK1) (Figure 8B). After the viral challenge, as expected, mice presented upregulation of genes related to the innate antiviral response (STAT2 and RNASEL). Interestingly, the expression of TLR2 and NLRP3 after challenge remained upregulated (1.03-fold-change ± 0.40 and 0.74-fold-change ± 0.25 respectively), in levels not statistically different from the ones triggered by immunization (1.18-fold-change ± 0.37 and 1.14-fold-change ± 0.40) (Figure 8B). Quantification of CLEC5A gene expression in animals revealed a slight increase following immunization compared with controls, which was also observed—at a higher level—in non-immunized animals challenged with SARS-CoV-2. However, in animals immunized with two doses of the vaccine and subsequently challenged with the virus, CLEC5A expression was significantly reduced compared with both vaccinated-only and non-immunized infected animals (Figure 8A–C).

### 3.8. Spike IgG Production and Polyfunctional Spike-Specific B and T Cells Activation After Immunization

**Figure 9:**
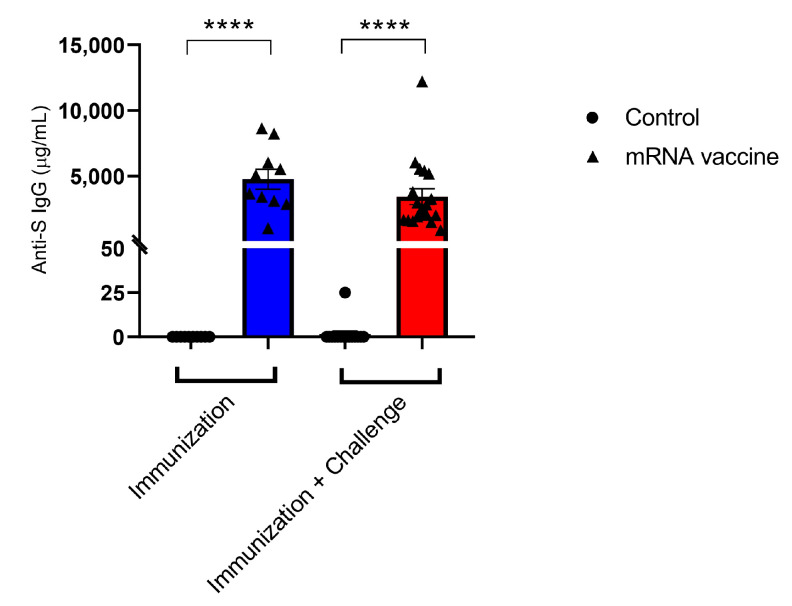
Spike IgG-detection during immunization and after viral challenge. The graphs represent the quantified Spike IgG production (µg/mL) in groups of mice: immunized non-virally challenged (blue column); immunized and virally challenged (red column); non-immunized and non-virally challenged (group control); non-immunized and virally challenged (group control). **** *p* < 0.0001.

**Figure 10:**
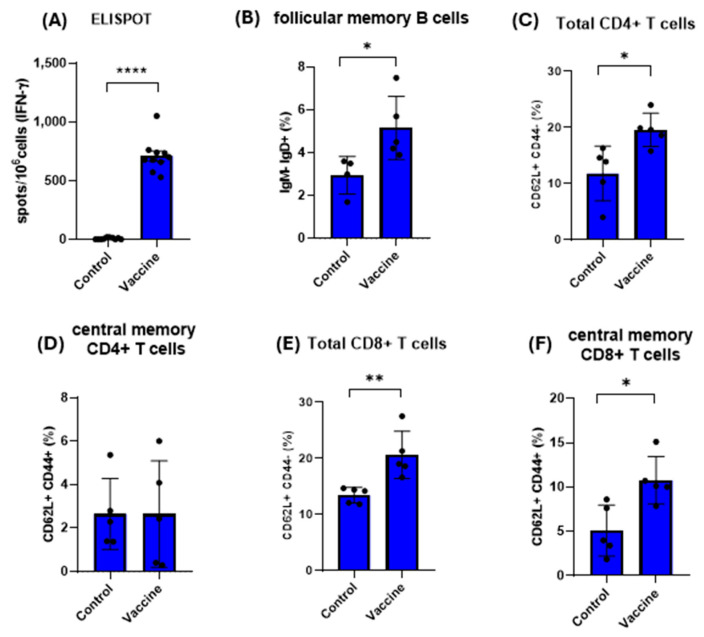
Cellular immune response evaluation after vaccination with two doses of bivalent commercial mRNA vaccine. The graphs represent the percentage expression of cells secreting IFN-gamma in ELISPOT data after vaccination (**A**), follicular memory B cells (**B**), total CD4+ T cells (**C**), central memory CD4+ T cells (**D**), total CD8+ T cells (**E**) and central memory CD8+ T cells (**F**) in the group of mice immunized. * *p* < 0.05; ** *p* < 0.01; **** *p* < 0.0001.

Immunogenicity was assessed through ELISA, ELISPOT and FACs analysis to evaluate whether any change in the adaptive immune response could be affected. The spike IgG antibodies were significantly elevated after immunization and after viral challenge (Figure 9). The splenocytes stimulated with spike from Omicron XBB.1.5 variant showed a significant increase in cells secreting IFN-gamma in ELISPOT data after vaccination (Figure 10A), and the percentage of follicular memory B cells was also increased after immunization (Figure 10B). Regarding memory T lymphocytes, it was observed that the percentage of total T cells was higher in vaccinated mice compared with non-vaccinated mice (control group) (Figure 10C–E). On the other hand, central memory CD8+ T cells were also elevated in immunized mice compared with the control group (Figure 10F).

### 3.9. In Silico Analysis of Spike Glycoprotein Residues Involved in Binding to Immune Cells and CLEC5A

**Figure 11:**
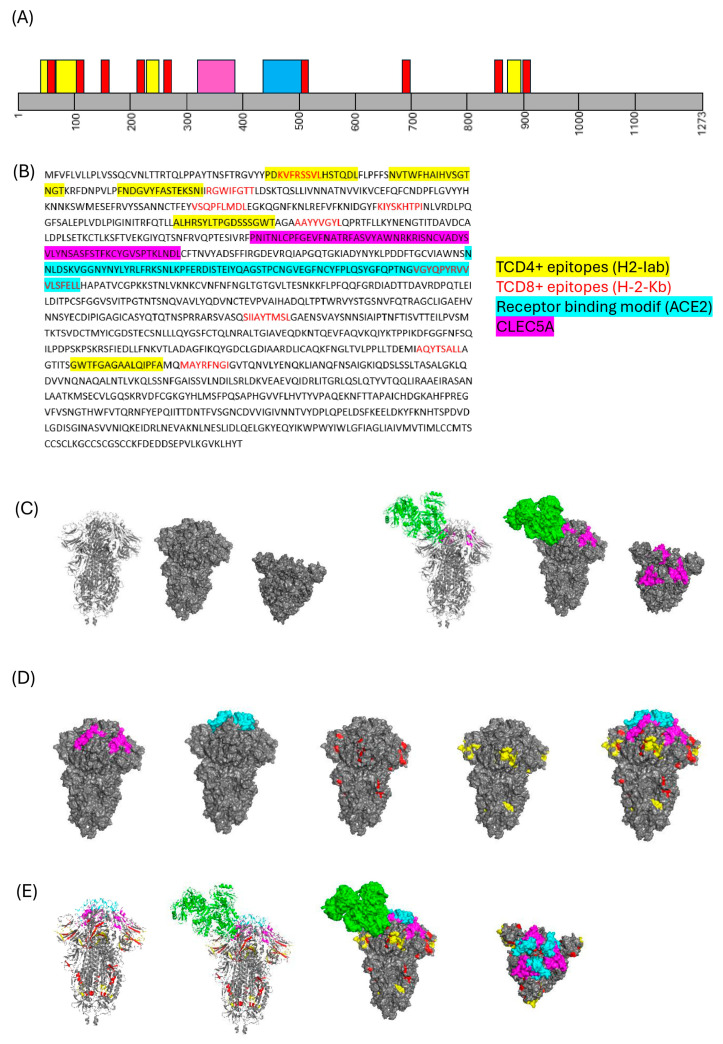
Molecular docking predicts the interactions between CLEC5A and spike glycoprotein domains with TCD4+ and TCD8+ epitopes in the mouse model study. (**A**) Domain organization of spike (gray) with TCD4+ (yellow) and TCD8+ (red) epitopes predicted for k-18-hACE2 mouse model, with binding sites with spike protein (pink). (**B**) Linear sequence of spike glycoprotein highlighting the TCD4+ and TCD8+ epitopes, as well as the receptor-binding motif (ACE2) (cyan) and CLEC5A site binding predicted. (**C**) Molecular docking prediction of CLEC5A molecule (green) and spike glycoprotein (pink), with horizontal and vertical views. (**D**) Left to right surface view obtained by PyMoL of closed spike glycoprotein (gray) highlighting the specific sites for CLEC5A, receptor motif ACE2, TCD8+ epitope, TCD4+ epitope and all of them together in the spike protein. (**E**) Overview of the CLEC5A receptor interaction with spike glycoprotein in cartoon mode and surface mode showing all the interactions predicted. Signal peptide: 1–12aa; S1-domain: 13–685aa; S2-domain: 686–1273aa; N-terminal domain (NTD): 13–305aa; supersite loops: N1:14–20aa; N3: 140–158aa; N5: 245–264aa; receptor-binding domain (RBD): 319–541aa; receptor-binding motif (ACE2): 437–508aa; furin cleavage sequence: 680–685aa; fusion peptide (FP): 788–806aa; heptad-repeat region 1 (HR1): 912–984aa; heptad-repeat region 2 (HR2): 1163–1213aa; transmembrane domain (TMD): 1214–1237aa; cytoplasmic domain: 1238–1273aa [20].

It was possible to observe that bioinformatics findings showed that T lymphocyte epitopes as well as B cell epitopes are not located in the same region of CLEC5A in the linear map of the spike glycoprotein amino acids sequence (Figure 11A,B). It was clearly demonstrated through molecular docking that the interaction with CLEC5A is between 330–390 residues of spike glycoprotein, while ACE-2 binds in 437–508 (Figure 11A–E). In addition, the local receptor binding modif (ACE-2) interaction with spike glycoprotein are distant from lymphocytes and CLEC5A predicted epitopes (Figure 11D,E).

## 4. Discussion

This study highlights the global impact of the COVID-19 pandemic and the rapid development of effective vaccines. With the approval and commercialization of COVID- 19 vaccines, an mRNA bivalent vaccine, including the Omicron BA.4/5 variant, was introduced in Brazil in 2023 [21]. Monitoring the immune response and inflammatory regulation following these vaccine formulations remains crucial. Our research emphasizes the importance of inflammation control in post-vaccination COVID-19 management by investigating C-type lectin mechanisms, particularly CLEC5A expression and inflammatory gene activation through in vitro, in vivo and in silico experiments [21,22].

The Pfizer-BioNTech bivalent mRNA vaccine efficiently delivered to THP-1 monocytes, with peak spike expression at 48 h, paralleling CLEC5A upregulation. This was accompanied by M1-like macrophage differentiation and robust CD86^+^ activation, in line with Zelkoski et al., 2025 [12]. In co-culture with human PBMCs from vaccinated subjects [17], THP-1 cells acted as functional APCs, driving inflammatory chemokine release and activation of both CD4^+^ and CD8^+^ T cells. Fluorescence microscopy showed that spike protein was expressed on the surface of THP-1 cells along with CD11a, a surface marker. These findings establish a mechanistic link between CLEC5A expression and T cell activation during SARS-CoV-2 immunization, consistent with our previous observations in yellow fever vaccination [9].

Our previous study with human samples revealed reduced CLEC5A expression in individuals who experienced natural SARS-CoV-2 infection after vaccination, compared to unvaccinated individuals with mild or severe COVID-19. Fully vaccinated individuals with a history of COVID-19 exhibited CLEC5A expression in immune cells, but at lower activation levels than those with severe disease. These findings support the role of CLEC5A in early innate immune activation, which may help mitigate SARS-CoV-2 infection and enhance immune responses following full vaccination [11]. Additionally, this present study demonstrated that inflammatory phenotype of monocyte-derived dendritic cells (CD11c+) can express CLEC5A after in vitro incubation with the mRNA vaccine using ex vivo blood from K-18-hACE-2 mice.

In an in vivo K18-hACE2 mouse model, the bivalent mRNA vaccine conferred protection against SARS-CoV-2 Gamma infection by reducing viral loads in nasal fluids, brain, and lungs, while preserving body weight and survival of immunized and challenged animals. The Gamma variant is capable of inducing mortality in susceptible animals, such as the K18-hACE2 mouse strain, thereby enabling the acquisition of more robust data on protection against infection using SARS-CoV-2 vaccines [23]. However, the use of the Omicron variant in the same mouse strain did not exhibit comparable pathogenicity [22,23]. Thus, it would not be a suitable variant for preclinical studies aimed at evaluating cause–effect relationships of vaccination, such as protective efficacy.

SARS-CoV-2 can disrupt systemic immune homeostasis, and effective vaccine functionality must address this issue, as seen in our follow-up clinical data and by others [21]. Previous studies indicated that C-type lectin receptors, such as CLEC5A on monocytes, play a significant role in the inflammatory response triggered by SARS-CoV-2 proteins [11]. Our findings confirmed the role of CLEC5A in the SARS-CoV-2 infection, as well as elevated levels of the inflammatory genes *TNFA* and *IL1B* after the viral challenge, alongside downregulation of antiviral genes, as seen by other groups with animal models [24,25].

Sung et al. (2022) demonstrated that CLEC5A, together with TLR2, is highly activated in SARS-CoV-2 infection, leading to potent inflammation in mouse models; both are critical markers in disease evolution and possible targets for therapies [26]. Our previous study also showed that CLEC5A was detected in monocytes from severe COVID-19 patients and in hamster models challenged with Delta and Omicron SARS-CoV-2 strains [11]. Here, in immunized mice, the CLEC5A and TLR2 expression were elevated after vaccination, but reduced in mice immunized and then challenged, reinforcing the concept of protection by an mRNA vaccine, also confirmed by robust specific spike-IgG, IFN-γ secretion, and memory B/T cell response in immunogenicity experiments [27,28,29].

In light of the above, our findings suggest that immunization could regulate CLEC5A, playing a role in inflammation control. This regulation was further supported by cytokine and inflammatory marker data from gene expression analyses. Our data can support mRNA vaccine development, serving as a model for preclinical studies to evaluate innate and adaptive immunity against several infectious diseases.

Molecular docking added information about the CLEC5A interaction with distinct regions of the spike glycoprotein compared with epitopes recognized by B- and T-cell peptides. In our previous study, it was demonstrated that spike protein subunit (S1) is able to interact with CLEC5A in a different manner, independently of ACE-2 receptor interaction with the host cells [11]. Here, we showed the localization of the spike protein to which CLEC5A binds. The CLEC5A residues interacting with the spike glycoprotein are not the same as the epitopes for immune cell activation. Although the range of immune response activation is large (250–800aa), the major activation site is under position 300aa for T cells and 400–500aa for neutralizing antibodies [30,31]. In addition, the other domains, such as the furin cleavage site, fusion peptide, and heptad-repeat region, are not involved in interactions with CLEC5A, as expected.

We acknowledge limitations common to animal studies, including the use of the highly inflammatory Gamma strain for viral challenge, despite the Omicron strain being a key component of the bivalent vaccine [29,30,31,32,33,34]. Our primary focus was to assess virus-induced inflammation and its control through immunization. Additionally, conducting post-challenge cell cultivation in a BSL-3 facility was limited due to the need for specialized training [22].

Regarding the in vitro assays, specifically the co-culture of PBMCs with THP-1 cells, we acknowledge the limitation of the small sample size (*n* = 5). Nevertheless, as preliminary data, these pilot studies are critical for generating initial insights and informing the design of more comprehensive investigations. Despite this limitation, the findings provide valuable information on APC-mediated peptide/protein recognition in previously vaccinated individuals.

While the activation of a robust adaptive immune response is not new to COVID-19 mRNA vaccines, the role of CLEC5A in immunization remains unclear. This study enhances our understanding of immune mechanisms, particularly the involvement of C-type lectins like CLEC5A in inflammation and memory responses. These insights are essential for improving vaccine efficacy against emerging variants and guiding next-generation vaccine development. Future research should further explore CLEC5A’s role and immune memory persistence across populations. Overall, this study underscores the importance of inflammation control and immune memory in effective disease management and vaccine strategies.

## Data Availability

The datasets used and/or analyzed during the current study are available from the corresponding author upon reasonable request.

## Supplementary Materials

The following supporting information can be downloaded at: https://www.mdpi.com/article/10.3390/v17091233/s1, Table S1: Primer pairs used in CLEC5A gene expression evaluation; Table S2: Primer pairs used in Fluidigm gene expression evaluation of genes related to innate immune response; Table S3: The information about the antibodies for mice and human samples used in the study. Figure S1: Gate strategy to get lymphocytes B and T by flow cytometry. (A) Gate strategy to get CD4+ T lymphocyte subpopulations. (B) Gate strategy to get CD8+ T lymphocyte subpopulations. (C) Gate strategy to get B lymphocyte subpopulations.
