# Correction: Galuzo et al. CLEC5A Activation in Inflammatory Monocytes: A Mechanism for Enhanced Adaptive Immunity Following COVID-19 mRNA Vaccination in a Preclinical Study. *Viruses* 2025, *17*, 1233

**DOI:** 10.3390/v17091281

**Published:** 2025-09-22

**Authors:** Renan Galuzo, Thiago Lazari Machado, Ryann de Souza Nascimento, Jorvan Ramos de Medeiros, Luciana Neves Tubarão, Jane Silva, Vanessa Pimenta Rocha, Tamiris Azamor, Felipe Soares Coelho, Andrea Marques Vieira da Silva, Lorenna Carvalho da Rosa, Juliana Fernandes Amorim da Silva, Renata Tourinho Santos, Rodrigo Müller, Carolina Baeta Salvador Várady, Ana Paula Dinis Ano Bom, Patricia Cristina da Costa Neves, Juliana Gil Melgaço

**Affiliations:** 1 Instituto de Tecnologia em Imunobiológicos, Bio-Manguinhos, Fundação Oswaldo Cruz, FIOCRUZ, Rio de Janeiro 21040-900, Brazil; renan.jorge@bio.fiocruz.br (R.G.); thiago.machado@bio.fiocruz.br (T.L.M.); ryann.nascimento@bio.fiocruz.br (R.d.S.N.); pcristina@bio.fiocruz.br (P.C.d.C.N.); 2 Biological Sciences Building, Kensington Campus, University of New South Wales, Sydney, NSW 2033, Australia

## Missing Institutional Review Board Statement

In the original publication [1], the Institutional Review Board Statement (IRBS) was not included in the Back Matter section in the standard format required by the journal. All experiments in the study were conducted in full compliance with the relevant ethical standards, and the corresponding ethics approvals were obtained prior to the study. The corrected statement is provided below.

The statement has now been added as follows:

**Institutional Review Board Statement:** The animal study protocol was approved by the Institutional Review Board (or Ethics Committee) of Fundação Oswaldo Cruz (protocol code P-6/20.3 (LW-17/20) on 6 July 2020 and renewed by protocol code 06/2024-3 (LBM-7/24) on 8 October 2024) by Bio-Manguinhos/Fundação Oswaldo Cruz. The protocol for the use of human samples was approved by the Institutional Review Board of Fundação Oswaldo Cruz (protocol code 34728920.4.0000.5262) on 3 November 2020.

The authors state that the scientific conclusions are unaffected. This correction was approved by the Academic Editor. The original publication has also been updated.
