# Supplementary material for: CLEC5A Activation in Inflammatory Monocytes: A Mechanism for Enhanced Adaptive Immunity Following COVID-19 mRNA Vaccination in a Preclinical Study
Source: Viruses. 2025 Sep 10;17(9):1233. doi: 10.3390/v17091233 (PMC12474447; doi:10.3390/v17091233)
Supplement: Supplementary file 1 [file viruses-17-01233-s001.zip › Supplementary Table S1.pdf]

Gene	Sense (Forward)	Antisense (Reverse)
CLEC5A	TTTTTCTGCTGTATTTCCCACA	ACGAAGCCATCATTACTTTTGC
PPIA	GCTTTTCGCCGCTTGCT	CTCGTCATCGGCCGTGAT
GAPDH	AACTTTGGCATTGTGGAAGG	GGAGACAACCTGGTCCTCAG

Supplementary Table S1. Primer pairs used in CLEC5A gene expression evaluation.
