# Supplementary material for: CLEC5A Activation in Inflammatory Monocytes: A Mechanism for Enhanced Adaptive Immunity Following COVID-19 mRNA Vaccination in a Preclinical Study
Source: Viruses. 2025 Sep 10;17(9):1233. doi: 10.3390/v17091233 (PMC12474447; doi:10.3390/v17091233)
Supplement: Supplementary file 1 [file viruses-17-01233-s001.zip › Supplementary Table S2.pdf]

Gene	Sense (Forward)	Antisense (Reverse)
ACTB	CATTGCTGACAGGATGCAGAAG	TGCTGGAAGGTGGACAGTGAGG
B2M	CAAGACCGTCTACTGGGATCG	TTGCTATTTCTTTCTGCGTGC
GAPDH	AACTTTGGCATTGTGGAAGG	GGAGACAACCTGGTCCTCAG
ACE2	TCCATTGGTCTTCTGCCATCCG	AGACCATCCACCTCCACTTCTC
ACO1	GCGCAAAGGACAAGAAAGCA	CCCCCAGCGGAATGTTTACT
AIM2	GTCCTCAAGCTAAGCCTCAGA	CACCGTGACAACAAGTGGAT
ASL	GGCAGAGACTAAAGGAGTGGCT	TCGACACTGGATTTCGCTGTGC
C1QA	AAAGGCAATCCAGGCAATATCA	TGGTTCTGGTATGGACTCTCC
C1QB	GGCAACCTGTGTGTGAATCTC	CTCTAGCTTCAAGACTACCCCA
C1QC	GTCTACTACACATCGCATACGG	ACGCTGTTGGAGCCCTCTA
C2	AGGTCTAGCTCACATCACACC	GCCGAGGGAAATAGATGCCATT
C3	CGCAACGAACAGGTGGAGATCA	CTGGAAGTAGCGATTCTTGGCG
C4A	GATGACAAGAACGTGAGTGTCC	AATCCAAAGTCCCTTTAGCCAC
C4B	ACTTCAGCAGCTTAGTCAGGG	GTCCTTTGTTTCAGGGGACAG
C5AR1	CCATTAGTGCCGACCGTTTCCT	CACGAAGGATGGAATGGTGAGG
C5AR2	GGAGACCTCTTCCTACTGGCTT	AGCCTACGGTAGACAGCAGAAG
CASP3	CTCGCTCTGGTACGGATGTG	TCCCATAAATGACCCCTTCATCA
CASP7	AAGACGGAGTTGACGCCAAG	CCGCAGAGGCATTTCTCTTC
CASP8	ATGGCTACGGTGAAGAACTGCG	TAGTTCACGCCAGTCAGGATGC
CASPASE1	TGGTCTTGTGACTTGGAGGA	TGGCTTCTTATTGGCACGAT
CCL2	CACTCACCTGCTGCTACTCA	GAGCTTGGTGACAAAAACTACAGC
CCL5	ATATGGCTCGGACACCACTC	CTTCGAGTGACAAACACGACTG
CFB	GCGAAAGTGTCAAGAAGGTGGC	ATCGGCTCCTTCGATGGTCTCT
CFH	AGGCTCGTGGTCAGAACAAC	GTTAGACGCCACCCATTTTCC
COX	TGAGCAACTATTCCAAACCAGC	GCACGTAGTCTTCGATCACTATC
CP	GAGTCTGGATGCTCAGTTGCCA	TATCCTCTGGCGAGGGCTTGTT
CXCL10	GTCTGAGTGGGACTCAAGGGAT	TCAACACGTGGGCAGGATAG
FECH	TCTTCCTGGACCGAGACCTCAT	CACCTCCGATTCTGCGATACTG
FHT1	CCGAGATGATGTGGCTCTGAA	CAGTCATCACGGTCTGGTTTCTTT
FTH1	TGAAGCTGGCATGGCAGAAT	AGCAGATGTTTTGGTGCAACT
FTL	GGTGAAGCTGCTGGATGAGT	CCTGTCGATTGTGCTCCACT
FX	CGTGGTGCATTTGAGGAACTTGG	GGCTTGTCTGCGAGGTCTTCAA
GADD45G	AAGTCCTGAATGTGGACCCTG	ATGGATCTGCAGCGCTATGT
GSDMD	TTCCAGTGCCTCCATGAATGT	GCTGTGGACCTCAGTGATCT
HAMP	CAGCACCACCTATCTCCATCAAC	CAGATGGGGAAGTTGGTGTCTC
HFE	GGACGAGACAAGGTTCACCTGT	GAAGATGGCACAGACGGTGACT
IFIT1	GCAGAGAGTCAAGGCAGGTT	TTGTGCATCCCCAATGGGTT
IFIT2	AGTTCTGGCCTTCTGCAGTT	GCTGTGTCAAAGCGCTCAAA
IFNA1	TTTCCCCTGACCCAGGAAGA	GGCTCTCCAGACTTCTGCTC
IFNB1	AACTCCACCAGCAGACAGTG	TGAGGACATCTCCCACGTCA
IFNG	TGGCTGTTTCTGGCTGTTAC	GATTTTCATGTCACCATCCTTTTG
IFNG G	TTCCCAGCAGCACAGAAACA	AATGGCACTTCAGCTGTGGA

IKBKB	GCAGACTGACATTGTGGACCTG	ATCTCCTGGCTGTCACCTTCTG
IKK1/CHUK	AGCAGCGTGCCATTGATCTC	GCTTCTTTGATGTTACTGAGGGC
IL10	ATTCCCTGGGTGAGAAGCTG	TAGACACCTTGGTCTTGGAGCTTA
IL18	TACAAGCATCCAGGCACAGC	GGCAGGAGTCCAGAAAGCAT
IL1B	TGCCACCTTTTGACAGTGATG	ATGTGCTGCTGCGAGATTTG
IL6	TGTACTCCAGGTAGCTATGG	GTTCTCTGGGAAATCGTGGA
IREB2	AGAAACGGACCTGCTCTTCCCA	CCTCTGTCTCAATGCCACCAAC
IRF3	GCGGGACTTCGTACATCTGG	TTCGGTAGGTTTTCCTGGGAG
IRF9	TGTCTGGAAGACTCGCCTAC	GCAACATCCATACGACCTCTCT
JAK1	ACGCTCCGAACCGAATCATC	GTGCCAGTTGGTAAAGTAGAACC
LRP2	CCAATGGACTCACTCTGGACCT	GAATGGAAGGCAGTGCTGATGAC
MMP2	CAAGGATGGACTCCTGGCACAT	TACTCGCCATCAGCGTTCCCAT
MMP7	CTTACCTCGGATCGTAGTGGA	CCCCAACTAACCCTCTTGAAGT
MMP9	GGACCCGAAGCGGACATTG	CGTCGTCGAAATGGGCATCT
MYD88	TCATGTTCTCCATACCCTTGGT	AAACTGCGAGTGGGGTCAG
NFKB	TGCTGATGGCACAGGACGAGAA	GTTGATGACGCCGAGGTACTGA
NFU1	GGAAGATGACGAAGTCGTGGCA	CACGATGCCATCTTCAAAGCCC
NLRP3	ATCAACAGGCGAGACCTCTG	GTCCTCCTGGCATACCATAGA
NOS1	TCCCAAGCCTATGCCAAGAC	CACAGCCGAATTTCTCCCCA
NOS2	GAGACAGGGAAGTCTGAAGCAC	CCAGCAGTAGTTGCTCCTCTTC
NOX1	AGTTTCTCTCCCGAAGGACCTC	GGTTAACCAGCCAGTTTCCCA
SPARC/ON	CACCTGGACTACATCGGACCAT	CTGCTTCTCAGTGAGGAGGTTG
PGE	AAGTCTGTCCTTGGTGCGAG	CGATGGAGAGGTAGCGTTCC
RNASEL	CATTGTGAACCTCCTGCTTAGT	CCGTGAATCCGTTCTCGTC
SDHB	TGCGGACCTATGGTGTTGGATG	CCAGAGTATTGCCTCCGTTGATG
SLC1	GGAAGAACCCTTTCCGCTTTGC	CACAGCGGAATGTAACTGGCAG
SLC40	CCATAGTCTCTGTCAGCCTGCT	CCATAGTCTCTGTCAGCCTGCT
STAT2	GTTACACCAGGTCTACTCACAGA	TGGTCTTCAATCCAGGTAGCC
TFR2	TCAGCGTGCTACACCTCAAAGC	CTCAATGAGGCTGACGAGAAGG
TLR2	CTCCAGGTTCTGATGTTGAAGT	GTTCAAGACTGCCCAGAGAATA
TLR4	AACTCAGCAAAGTCCCTGATG	ATTGTTTCAATTTCACACCTGGAT
TLR9	AGAGACCCTGGTGTGGAAC	CTTCGACGGAGAACCATGT
TRAF6	TTTCCCTGACGGTAAAGTGCCC	ACCTGGCACTTCTGGAAAGGAC
TRFC	GAAGTCCAGTGTGGGAACAGGT	CAACCACTCAGTGGCACCAACA
TYK2	GACTTTGTGTGCGATGTGGAT	AGCCATCTTGGAAGACAGCAA

Supplementary Table S2. Primer pairs used in Fluidigm gene expression evaluation of genes related to innate immune response.
