# Supplementary material for: CLEC5A Activation in Inflammatory Monocytes: A Mechanism for Enhanced Adaptive Immunity Following COVID-19 mRNA Vaccination in a Preclinical Study
Source: Viruses. 2025 Sep 10;17(9):1233. doi: 10.3390/v17091233 (PMC12474447; doi:10.3390/v17091233)
Supplement: Supplementary file 1 [file viruses-17-01233-s001.zip › Supplementary Table S3.pdf]

Supplementary Table S3: The information about the antibodies for mice and human samples used in the study.

**Murine antibodies**		
Panel 1		
Marker	Fluorophore	Catalog
CD4	PERCPCy5.5	550954
CD8a	PECy 7	552877
CD19	APC	550992
CD44	BV605	563058
CD62L	BV510	563117
B220	FITC	553087
IgM	PE	553409
IgD	APC-H7	565348
Viability dye *LIVE/DEAD*	Ultraviolet (UV)	L23105A
Panel 2		
Marker	Fluorophore	Catalog
Ly6C	BV421	562727
CX3CR1	PECy7	567820
CCR2	BV711	747964
CD11c	APC	561119
CLEC5A	PE	FAB1639P
Viability dye *LIVE/DEAD*	Ultraviolet (UV)	L23105A
**Human antibodies**		
Panel THP-1 (1)		
Marker	Fluorophore	Catalog
CD11b	APC	550019
CD38	PECy7	560677
CD86	PECF594	562390

CD69	BV421	562884
Viability dye *LIVE/DEAD*	Ultraviolet (UV)	L23105A
Panel THP-1 (2)		
Marker	Fluorophore	Catalog
CLEC5A	PE	FAB2384P
Sars-CoV-2 *Spike* S1 *Subunit*	AF488	FAB105403G
Viability dye *LIVE/DEAD*	Ultraviolet (UV)	L23105A
Panel THP-1 (Fluorescence microscopy)		
Marker	Fluorophore	Catalog
CD11a	PE	555380
Sars-CoV-2 *Spike* S1 *Subunit*	AF488	FAB105403G
Panel PBMC		
Marker	Fluorophore	Catalog
CD4	BV421	562842
CD3	FITC	349201
CD134 (OX40)	BV711	563664
CD69	BV421	562884
Viability dye *LIVE/DEAD*	Ultraviolet (UV)	L23105A
