# Supplementary figures and images for: CLEC5A Activation in Inflammatory Monocytes: A Mechanism for Enhanced Adaptive Immunity Following COVID-19 mRNA Vaccination in a Preclinical Study
Source: Viruses. 2025 Sep 10;17(9):1233. doi: 10.3390/v17091233 (PMC12474447; doi:10.3390/v17091233)

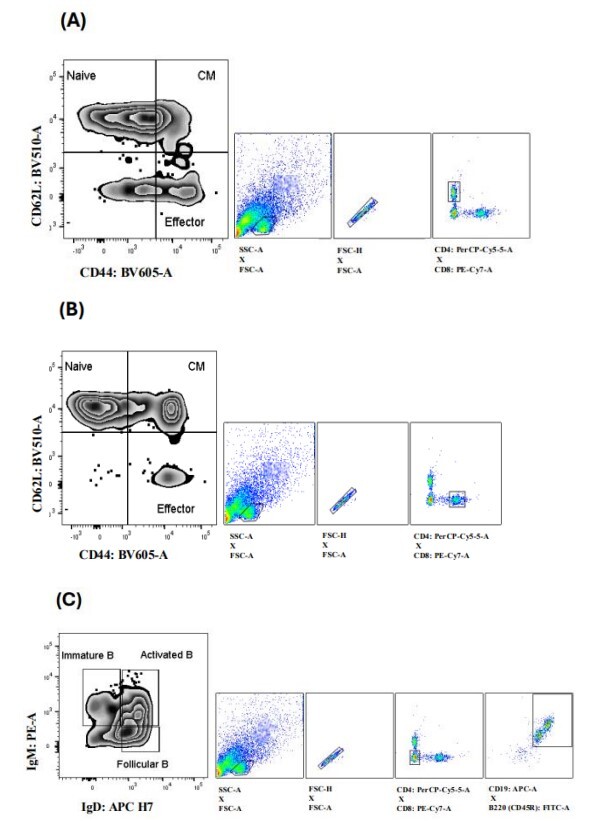

Supplement: Supplementary file 1 [file viruses-17-01233-s001.zip › Supplementary Figure S1.jpg]
